# Comparison of clinical outcomes in patients with refractory ascites treated with either TIPS, tunneled peritoneal catheter, or ascites pump

**DOI:** 10.1097/HC9.0000000000000620

**Published:** 2025-01-16

**Authors:** Sarah L. Schütte, Anja Tiede, Jim B. Mauz, Hannah Rieland, Martin Kabelitz, Robin Iker, Nicolas Richter, Bernhard Meyer, Benjamin Heidrich, Heiner Wedemeyer, Benjamin Maasoumy, Tammo L. Tergast

**Affiliations:** 1 Department of Gastroenterology, Hepatology, Infectious Diseases and Endocrinology, Hannover Medical School, Hannover, Germany; 2 German Center for Infection Research (DZIF), Hannover-Braunschweig, Germany; 3 Department of Abdominal and Transplant Surgery, Hannover Medical School, Hannover, Germany; 4 Department of Diagnostic and Interventional Radiology, Hannover Medical School, Hannover, Germany; 5 Excellence Cluster Resist, Hannover Medical School, Hannover, Germany

**Keywords:** cirrhosis, peritonitis, portal hypertension

## Abstract

**Background:**

Refractory ascites (RA) remains a serious complication in patients with cirrhosis. Currently, the insertion of a TIPS is considered the standard of care in these patients. To achieve symptom control in those with TIPS contraindications, tunneled peritoneal catheters (PeCa) or ascites pumps were introduced. However, data comparing the available treatment options are scarce. This study aims to compare outcomes among patients with RA treated either with TIPS, PeCa, or ascites pump.

**Methods:**

All patients with RA and cirrhosis treated at Hannover Medical School between 2009 and 2023 were evaluated. Endpoints included mortality, acute kidney injury (AKI), hyponatremia, peritonitis, and rehospitalization rate. Propensity score matching was conducted to adjust for group differences.

**Results:**

First, 31 patients with ascites pump were compared to 62 patients with a PeCa after propensity score matching. There were no differences regarding mortality nor incidences of AKI, hyponatremia, or rehospitalization. However, incidences of peritonitis and explantation were lower in those with ascites pump (HR 0.32, 95% CI: 0.15–0.70, and HR 0.32, 95% CI: 0.14–0.71, respectively). Second, 35 ascites pump patients were matched with 70 individuals with TIPS. No differences regarding mortality or peritonitis incidence were observed. Ascites pump patients showed higher incidences of AKI (HR 4.55, 95% CI: 2.53–8.18) and hyponatremia (HR 4.13, 95% CI: 2.08–8.22). Last, 129 patients with TIPS were compared to 129 with PeCa. Mortality was comparable, while incidences of AKI (HR 5.01, 95% CI: 3.36–7.47), hyponatremia (HR 4.64, 95% CI: 3.03–7.12), and peritonitis (HR 2.19, 95% CI: 1.41–3.41) were higher in those with PeCa.

**Conclusions:**

While ascites pump was associated with lower incidences of device infections and explantations, TIPS was associated with the lowest incidence of clinical complications in patients with RA.

## Graphical abstract

**Figure:**
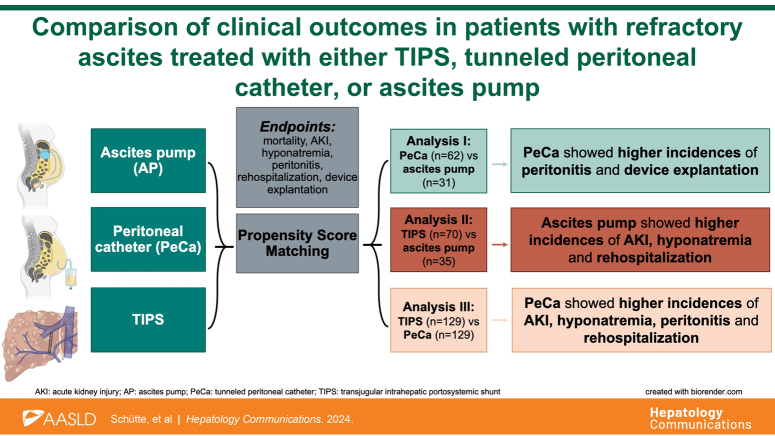

## INTRODUCTION

The development of clinically significant portal hypertension indicates a watershed moment in the natural course of advanced chronic liver disease (ACLD).^1^ In the setting of clinically significant portal hypertension, the risk of decompensation is drastically increased.^2^ Here, the development of ascites is the most frequent type of first decompensation. Large-volume paracentesis (LVP), plasma expansion with albumin and administration of diuretics are the current standard of care (SOC) to achieve symptom control.^3^ In some patients, reoccurrence of ascites cannot be prevented with diuretics due to either the ineffectiveness of even extensive doses of diuretics or due to diuretic-derived complications that ultimately lead to the cessation of the respective treatment. This clinical phenotype is classified as refractory ascites (RA) and defines the final stage of ACLD.^4^ Overall, prognosis is significantly worsened compared to other stages of ACLD and complications such as acute kidney injury (AKI) or hyponatremia are frequent, leading to hospitalization and an overall decreased quality of life.^4^,^5^,^6^,^7^

If liver transplantation is not available, implantation of a TIPS has been established as an effective treatment option that decreases the portal pressure and often leads to the resolution of ascites. However, some patients have contraindications such as repeated HE without a precipitating event or advanced chronic heart failure, which ultimately are contraindications for TIPS implantation. In these patients, repeated LVP with albumin substitution remains the recommended SOC, resulting in frequent hospitalizations for patients. To enable home-based treatment for RA in those with contraindications for TIPS, low-flow devices, ie, an ascites pump and a tunneled peritoneal catheter (PeCa), have been introduced into clinical practice.^5^ The ascites pump continuously delivers ascites out of the peritoneal cavity directly into the bladder, while PeCa is a direct permanent drainage system that allows ascites drainage via connectable drainage bags. Both devices achieve symptom control. While there are studies available comparing ascites pump or PeCa to SOC, there are no comparative studies between the 2 devices regarding important clinical endpoints.^5^,^8^,^9^ Furthermore, only very limited evidence is available comparing ascites pump and TIPS and no data are available that analyzed outcomes between patients with PeCa and TIPS implantation.^10^ Hence, this study compared the clinical course of patients with RA who underwent either ascites pump, PeCa, or TIPS implantation.

## METHODS

### Study cohort

This retrospective cohort study was performed at Hannover Medical School, Germany. Data were collected between 2012 and 2023. All consecutive patients who were treated for RA and received either TIPS, ascites pump, or PeCa were selected for this study. Overall, 918 patients were considered for this study. Patients without liver cirrhosis or RA, underlying malignant diseases, prior organ transplantation, and congenital immune dysfunction diseases were excluded from this study (Supplemental Figure S1, http://links.lww.com/HC9/B863). Data regarding TIPS, PeCa, and ascites pump implantation have been described.^11^,^12^,^13^

### Data assessment and study endpoints

Information regarding study patients and laboratory values was collected automatically via the Clinical Information system with subsequent manual validation. Data concerning the patients’ clinical course were obtained from their medical records. In this study, patients were followed for 1 year after device implantation. Study endpoints consisted of mortality, incidence of AKI, hyponatremia, incidence of peritonitis, rehospitalization, and need for device explantation. Hyponatremia was defined as serum sodium<130 mmol/L. If patients presented with hyponatremia at baseline, analysis for hyponatremia started when sodium was documented above 130 mmol/L. Diagnosis of AKI was based on current guidelines.^14^

### Study design

Overall, 3 different cohorts were analyzed and compared in this study. Hence, 3 different analyses were conducted. Due to expected baseline differences between the respective study cohorts, propensity score matching (PSM) was conducted. Finally, 38 patients with an ascites pump, 187 patients with a PeCa, and 243 patients with TIPS insertion were included in this study (Supplemental Figure S1, http://links.lww.com/HC9/B863).

First, patients who received PeCa implantation were compared to those who received an ascites pump (analysis I). Second, patients who underwent TIPS insertion were matched with patients in whom an ascites pump was implanted (analysis II). For analyses I+II, PSM was conducted in a 2:1 manner to achieve a higher statistical power. Third, those with PeCa were compared to patients with TIPS. Here, 1:1 PSM was applied (analysis III).

### Statistics

Data were analyzed using SPSS Statistics Version 28 (IBM) and R Version 4.3.2 (R Project for Statistical Computing, packages MatchIt, Rcmdr, Rcmdr.Plugin EZR, Cmprsk, Plotly).^15^,^16^ PSM was conducted using a Nearest Neighbor Matching with a caliper of 0.15. Patients were matched for preprocedure values of the Freiburg Index for post-TIPS survival (FIPS) and international normalized ratio, to adjust for liver function and the severity of the liver disease.^17^,^18^ Additionally, intake of diuretics before device implantation was also implemented in the PSM to adjust for severity therapy of the ascites. Last, history of spontaneous bacterial peritonitis (SBP) was included in PSM to adjust for susceptibility to infections. Standardized mean differences were used for evaluating balance prior to and following PSM. After PSM, a standardized mean difference of 0.10 or below was considered successful (Supplemental Table S1A–C, http://links.lww.com/HC9/B864, Supplemental Figure S2A–C, http://links.lww.com/HC9/B863). Categorial variables are given as total numbers and proportions. To calculate group differences, the Cochran-Mantel-Haenszel test was used in the 2:1 matched cohorts and the McNemar test for 1:1 matched cohorts. Continuous variables are presented as mean with SD. Here, the ANOVA test was applied in the 2:1 matched cohorts, while paired *t* testing was used in the 1:1 matched cohorts. Kaplan-Meier curves were used to illustrate survival or incidences in the respective analyses. Time-to-event data were analyzed using univariable and multivariable Fine and Gray competing risk analysis, treating death/liver transplantation (LTx) as competing events. In terms of LTx-free survival, death was the event of interest, while LTx was considered the competitor.

### Ethics

This study was approved by the local ethics committee (Ethics approval numbers Nr. 9428_BO_K_2020, Nr. 10073_BO_K_2021 and Nr. 8498_BO_S_2019). It followed the Declaration of Helsinki and Istanbul.^19^ Patients had to declare their informed consent for analysis of their data and participation in this study.

## RESULTS

As mentioned above, 38 patients with an ascites pump, 187 patients with a PeCa and 243 patients with TIPS insertion were included in this study. There were significant differences regarding baseline characteristics between the respective groups (Supplemental Table S2A–C, http://links.lww.com/HC9/B864).

### Analysis I (PeCa vs. ascites pump)

Baseline characteristics differed significantly prior to PSM (Supplemental Table S2A, http://links.lww.com/HC9/B864). After PSM, 62 patients with PeCa were compared with 31 individuals who received ascites pump treatment (Table 1, Supplemental Figure S1, http://links.lww.com/HC9/B863). Standardized mean differences of the matched parameters were comparable after matching (Supplemental Table S1A–C, http://links.lww.com/HC9/B864). The majority of patients were male (PeCa: n=40 [65%] vs. ascites pump: n=20 [65%], *p*=1.00) and had alcohol-associated liver disease (PeCa: n=20 [32%] vs. ascites pump: n=14 [45%], *p*=0.33). Furthermore, patients had comparable MELD (PeCa, mean [SD]: 15 [5] vs. ascites pump, mean [SD]: 14 [3], *p*=0.17) and FIPS-Scores (PeCa, mean [SD]: 0.28 [0.77] vs. ascites pump, mean [SD]: 0.28 [0.48], *p*=0.96) (Table 1).

**TABLE 1:** Baseline characteristics after PSM for analysis I

Parameter	PeCa (n=62)	Ascites pump (n=31)	*p*-value
Sex—male/female, n (%)	40 (65)/22 (35)	20 (65)/11 (35)	1.00
Underlying disease, n (%)			
ALD	20 (32)	14 (45)	0.33
MetALD	6 (10)	3 (10)	1.00
MASLD	5 (8)	4 (13)	0.71
Viral	10 (16)	5 (16)	1.00
Cryptogenic	7 (11)	0 (0)	0.13
Other	16 (26)	5 (16)	0.433
Age, mean (SD), y	63 (11)	57 (10)	0.01
MELD, mean (SD)	15 (5)	14 (3)	0.17
FIPS, mean (SD)	0.28 (0.77)	0.28 (0.48)	0.96
Creatinine, mean (SD), mg/dL	1.66 (0.74)	1.49 (0.39)	0.23
Bilirubin, mean (SD), mg/dL	1.78 (2.06)	1.57 (1.49)	0.61
INR, mean (SD)	1.29 (0.24)	1.28 (0.16)	0.77
Leukocyte count, mean (SD), ×10^3^/μL	6.7 (4.9)	6.0 (2.6)	0.48
Sodium, mean (SD), mmol/L	132 (18)	135 (5)	0.45
ALT, mean (SD), IU/mL	30 (40)	28 (14)	0.76
Albumin, mean (SD), g/L	30 (7)	28 (6)	0.19
Platelet count, mean (SD), ×10^3^/μL	129 (108)	116 (51)	0.54
Diuretics	54 (87)	27 (87)	1.00
Loop diuretics, n (%)	50 (81)	24 (77)	0.93
Mean dosage of furosemide, mean (SD), mg	91 (75)	93 (77)	0.92
Aldosteron antagonists, n (%)	39 (63)	24 (77)	0.24
Mean dosage of spironolactone, mean (SD), mg	109 (86)	124 (95)	0.52
History of SBP, n (%)	18 (29)	10 (32)	0.94
Contraindications against TIPS implantation, n (%)			
Risk for HE	29 (47)	17 (55)	0.61
Heart failure	17 (27)	2 (7)	0.04
Technically impossible	6 (10)	5 (16)	0.57
Other	11 (18)	6 (19)	0.93

Abbreviations: ALD, alcohol-associated liver disease; FIPS, Freiburg index of post-TIPS survival; INR, international normalized ratio; MASLD, metabolic dysfunction–associated steatotic liver disease; MetALD, metabolic and alcohol-associated liver disease; PeCa, tunneled peritoneal catheter; PSM, propensity score matching; SBP, spontaneous bacterial peritonitis.

**FIGURE 1:**
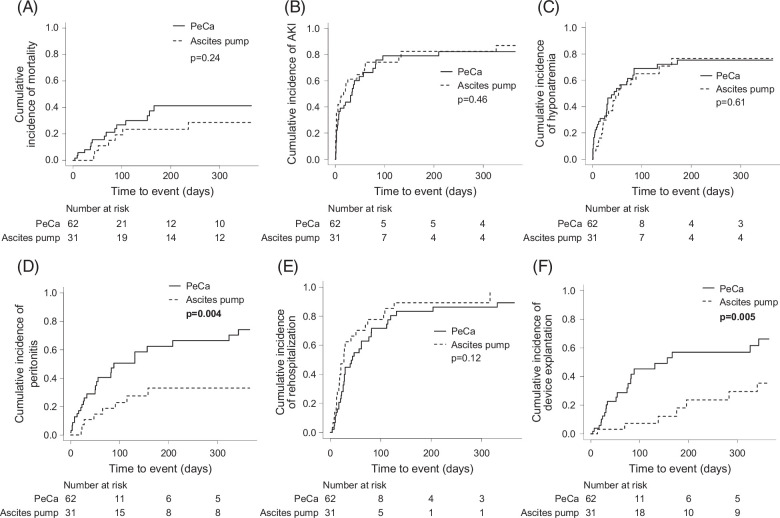
Kaplan-Meier curves illustrating the cumulative incidences of mortality (A), AKI (B), hyponatremia (C), peritonitis (D), rehospitalization (E), and device explantation (F) in patients with PeCa compared to those with ascites pump in 1 year. Death and liver transplantation were considered as competing events. Attached *p*-values are the result of univariable competing risk analysis. Abbreviations: AKI, acute kidney injury; PeCa, tunneled peritoneal catheter.

Regarding study endpoints, 365-day mortality (PeCa 41% vs. ascites pump 29%, HR 0.59, 95% CI: 0.25–1.41, *p*=0.24), the incidence of AKI (PeCa 82% vs. ascites pump 87%, HR 1.21, 95% CI: 0.73–1.99, *p*=0.46), hyponatremia<130 mmol/L (PeCa 75% vs. ascites pump 77%, HR 0.87, 95% CI: 0.52–1.47, *p*=0.61), and rehospitalization (PeCa 89% vs. ascites pump 100%, HR 1.49, 95% CI: 0.90–2.46, *p*=0.12) did not differ between ascites pump and PeCa patients (Figure 1A–C, E). Of note, 8% of the patients with PeCa and 9% of the patients with an ascites pump underwent LTx within the first year following device implantation, while 41% of patients with PeCa and 29% of patients with ascites pump died within 365 days. Yet, the incidence of peritonitis (PeCa 74% vs. ascites pump 33%, HR 0.32, 95% CI: 0.15-0.70, *p*=0.004) and the need for explantation (PeCa 66% vs. ascites pump 35%, HR 0.32, 95% CI: 0.14–0.71, *p*=0.005) were significantly lower in patients treated with the ascites pump (Figure 1D, F). Due to significant differences in the patients´ age after PSM we chose to conduct a multivariable competing risk analysis. The findings remained similar after adjusting for age (Supplemental Table S3A, http://links.lww.com/HC9/B864). Infection was the main reason for explantation in both groups (PeCa n=12 [55%], ascites pump n=4 [57%]; Supplemental Table S4, http://links.lww.com/HC9/B864). The most common reasons for rehospitalization were renal impairment, ascites, and dysfunction of the catheter for patients with ascites pump (n=4 [16%]; n=3 [12%]; n=5 [20%]; respectively; Supplemental Table S5A, http://links.lww.com/HC9/B864). Patients with PeCa were mostly readmitted to the hospital due to peritonitis (n=10 (29%); Supplemental Table S5A, http://links.lww.com/HC9/B864). *Enterococcus spp*. (ascites pump n=2 [50%], PeCa n=7 [23]) and *Staphylococcus spp.* (ascites pump n=1 [25%], PeCa n=15 [48%]) were found in ascites from both groups during peritonitis, whereas Acinetobacter spp. (n=3 [10%]) could only be detected in ascites from patients with PeCa (Supplemental Table S6A, http://links.lww.com/HC9/B864; Supplemental Figure S3, http://links.lww.com/HC9/B863).

### Analysis II (TIPS vs. ascites pump)

In total, 70 patients who underwent TIPS insertion were compared to 35 with ascites pump after PSM. No statistically significant differences regarding MELD (TIPS, mean [SD]: 13 [4] vs. ascites pump, mean [SD]: 13 [3], *p*=0.87) or FIPS (TIPS, mean [SD]: 0.03 [0.66] vs. ascites pump, mean [SD]: 0.05 [0.65], *p*=0.80) were documented. Most patients were male (TIPS: n=39 (56%) vs. ascites pump: n=24 (69%), *p*=0.29) and had alcohol-associated liver disease (TIPS: n=36 [51%] vs. ascites pump: n=16 [48%] *p*=0.73) (Table 2).

**TABLE 2:** Baseline characteristics after PSM for analysis II

Parameter	TIPS (n=70)	Ascites pump (n=35)	*p*
Sex—male/female, n (%)	39 (56)/31 (44)	24 (69)/11 (31)	0.29
Underlying disease, n (%)			
ALD	36 (51)	16 (48)	0.73
MetALD	9 (13)	3 (9)	0.75
MASLD	9 (13)	4 (11)	0.92
Viral	6 (9)	6 (17)	0.33
Cryptogenic	5 (7)	0	0.26
Other)	6 (9)	6 (17)	0.33
Age, mean (SD), y	63 (10)	57 (10)	0.008
MELD, mean (SD)	13 (4)	13 (3)	0.87
FIPS, mean (SD)	0.03 (0.66)	0.05 (0.65)	0.80
Creatinine, mean (SD), mg/dL	1.50 (0.73)	1.41 (0.43)	0.48
Bilirubin, mean (SD), mg/dL	1.03 (0.68)	1.40 (1.43)	0.08
INR, mean (SD)	1.24 (0.18)	1.25 (0.16)	0.83
Leukocyte count, mean (SD), ×10^3^/μL	5.8 (2.3)	5.7 (2.1)	0.92
Sodium, mean (SD), mmol/L	134 (5)	135 (6)	0.91
ALT, mean (SD), IU/mL	26 (16)	27 (14)	0.71
Albumin, mean (SD), g/L	30 (7)	29 (5)	0.40
Platelet count, mean (SD), ×10^3^/μL	133 (72)	138 (89)	0.74
Diuretics, n (%)	62 (89)	31 (91)	0.91
Loop diuretics, n (%)	53 (76)	29 (83)	0.56
Mean dosage of furosemide, mean (SD), mg	89 (42)	87 (72)	0.83
Aldosteron antagonists, n (%)	59 (84)	28 (80)	0.79
Mean dosage of spironolactone, mean (SD), mg	170 (108)	134 (95)	0.14
History of SBP, n (%)	18 (26)	10 (29)	0.94

Abbreviations: ALD, alcohol-associated liver disease; FIPS, Freiburg index of post-TIPS survival; INR, international normalized ratio; MASLD, metabolic dysfunction–associated steatotic liver disease; MetALD, metabolic and alcohol-associated liver disease; PSM, propensity score matching; SBP, spontaneous bacterial peritonitis.

**FIGURE 2:**
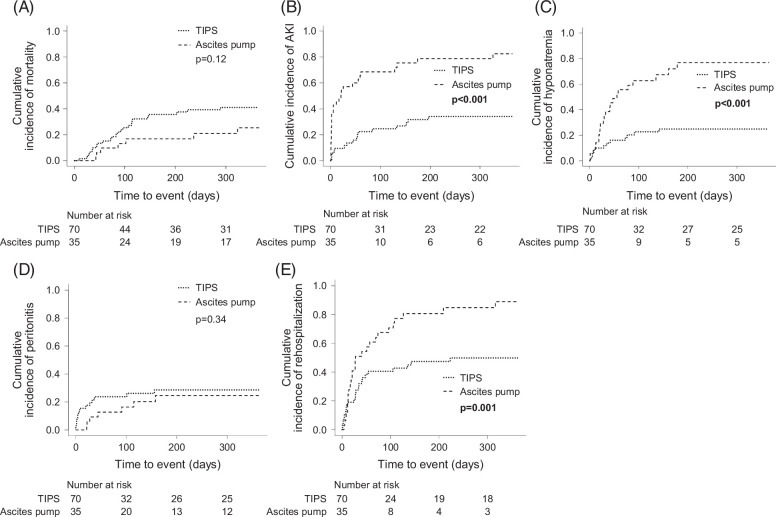
Kaplan-Meier-curves illustrating the cumulative incidences of mortality (A), AKI (B), hyponatremia (C), peritonitis (D), and rehospitalization (E) in patients with ascites pump and patients with TIPS in one year. Death and liver transplantation were considered competing events. Attached *p-*values are the result of univariable competing risk analysis. Abbreviation: AKI, acute kidney injury.

No significant differences in terms of mortality nor incidence of peritonitis were observed between the analyzed groups (TIPS 41% vs. ascites pump 25%, HR 0.52, 95% CI: 0.23–1.19, *p*=0.12; TIPS 29% vs. ascites pump 25%, HR 0.66, 95% CI: 0.29–1.54, *p*=0.34, respectively; Figure 2A, D). Notably, transplantation rates in the first 365 days were 2% in the TIPS group and 8% in the ascites pump cohort. The cumulative incidence of mortality within one year was 41% in the TIPS cohort, and 25% in the ascites pump cohort. However, the risk of developing an AKI (TIPS 34% vs. ascites pump 82%, HR 4.55, 95% CI: 2.53–8.18, *p*<0.001), hyponatremia (TIPS 25% vs. ascites pump 77%, HR 4.13, 95% CI: 2.08–8.22, *p*<0.001), and rehospitalization (TIPS 50% vs. ascites pump 89%, HR 2.49, 95% CI: 1.44–4.31, *p*=0.001) was significantly higher in patients with an ascites pump (Figure 2B, C, E). The mean age in both cohorts differed after PSM. Hence, we applied a multivariable competing risk analysis and adjusted for age. All findings persisted after adjustment for age (Supplemental Table S3B, http://links.lww.com/HC9/B864). The main reason for rehospitalization was ascites requiring therapy and HE in patients with TIPS (n=9 [39%] and n=9 [39%], respectively, Supplemental Table S5B, http://links.lww.com/HC9/B864) and renal impairment, ascites, dysfunction of the catheter, or HE in patients with ascites pump (n=4 [15%]; n=4 [15%]; n=4 [15%]; n=4 [15%]; respectively, Table S5B, http://links.lww.com/HC9/B864). Cultural germ detection was only successful in a total of six cultures cultivated with ascites from peritonitis. In those, *Enterococcus spp*. (TIPS n=1 [33%], ascites pump n=1 [33%]) and *Escherichia spp*. (TIPS n=1 [33%], ascites pump n=1 [33%]) were found in patients from both groups (Supplemental Table S6B, http://links.lww.com/HC9/B864).

### Analysis III (PeCa vs. TIPS)

After PSM, 129 individuals with PeCa implantation and 129 patients with TIPS implantation were compared. Again, baseline factors are displayed in Table 3. MELD score (PeCa, mean [SD]: 15 [4] vs. TIPS: 15 [4], *p*= 0.18) and FIPS (PeCa, mean [SD]: 0.24 [0.75] vs. TIPS, mean [SD]: 0.22 [0.64], *p*= 0.74) did not differ significantly between both groups.

**TABLE 3:** Baseline characteristics after PSM for analysis III

Parameter	PeCa (n=129)	TIPS (n=129)	*p*
Sex—male/female, n (%)	89 (69)/40 (31)	80 (62)/49 (38)	0.30
Underlying disease, n (%)			
ALD	50 (39)	55 (43)	0.62
MetALD	14 (11)	20 (16)	0.38
MASLD	12 (9)	14 (11)	0.84
Viral	13 (10)	11 (9)	0.83
Cryptogenic	13 (10)	9 (7)	0.52
Other	29 (23)	20 (16)	0.20
Age, mean (SD), y	56 (19)	60 (11)	0.02
MELD, mean (SD)	15 (4)	15 (4)	0.18
FIPS, mean (SD)	0.24 (0.75)	0.22 (0.64)	0.74
Creatinine, mean (SD), mg/dL	1.54 (0.73)	1.67 (0.94)	0.19
Bilirubin, mean (SD), mg/dL	1.92 (1.95)	1.30 (1.01)	0.001
INR, mean (SD)	1.34 (0.24)	1.34 (0.22)	0.81
Leukocyte count, mean (SD), ×10^3^/μL	6.6 (4.1)	6.2 (3.7)	0.41
Sodium, mean (SD), mmol/L	133 (13)	135 (5)	0.05
ALT, mean (SD), IU/mL	32 (35)	36 (124)	0.74
Albumin, mean (SD), g/L	30 (7)	29 (7)	0.62
Platelet count, mean (SD), ×10^3^/μL	122 (88)	130 (75)	0.42
Diuretics, n (%)	100 (78)	100 (78)	1.00
Loop diuretics, n (%)	89 (69)	88 (69)	1.00
Mean dosage of furosemide, mean (SD), mg	81 (52)	114 (101)	0.02
Aldosteron antagonists, n (%)	76 (59)	88 (68)	0.10
Mean dosage of spironolactone, mean (SD), mg	97 (71)	146 (98)	0.002
History of SBP	58 (45)	61 (47)	0.80

Abbreviations: ALD, alcohol-associated liver disease; FIPS, Freiburg Index of post-TIPS survival; INR, international normalized ratio; MASLD, metabolic dysfunction–associated steatotic liver disease; MetALD, metabolic and alcohol-associated liver disease; PeCa, tunneled peritoneal catheter; PSM, propensity score matching; SBP, spontaneous bacterial peritonitis.

**FIGURE 3:**
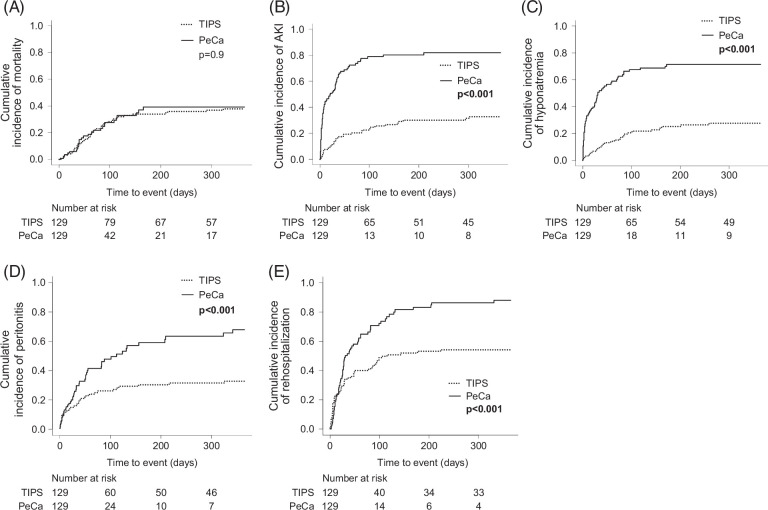
Kaplan-Meier-curves illustrating the cumulative incidences of mortality (A), AKI (B), hyponetremia (C), peritonitis (D), and rehospitalization (E) in patients with PeCa and those treated with TIPS in 1 year. Death and liver transplantation were considered competing events. The attached *p-*values are the result of univariable competing risk analysis. Abbreviations: AKI, acute kidney injury; PeCa, tunneled peritoneal catheter.

Three hundred sixty-five-day mortality was comparable between both groups (TIPS 38% vs. PeCa 39%, HR 1.03, 95% CI: 0.64–1.66, *p*=0.9) (Figure 3A). LTx rates within the first year after implantation were 5% for the TIPS group and 12% for the PeCa group. The mortality rates were comparable, with 38% of TIPS patients and 39% of patients with PeCa passing away within the first year after implantation. Patients with PeCa had a significantly higher risk of developing an AKI and hyponatremia (TIPS 33% vs. PeCa 82%, HR 5.01, 95% CI: 3.36–7.47, *p*<0.001; TIPS 28% vs. PeCa 71%, HR 4.64, 95% CI: 3.03–7.12, *p*<0.001, respectively; Figure 3B-C), as well as a higher incidence of peritonitis and rehospitalization (TIPS 33% vs. PeCa 68%, HR 2.19, 95% CI: 1.41–3.41, *p*<0.001 and TIPS 54% vs. PeCa 88%, HR 1.94, 95% CI: 1.35–2.80, *p*<0.001, respectively; Figure 3D, E). Due to significant differences in baseline age and dosages of loop diuretics and aldosterone antagonists prior to device implantation between the respective cohorts after PSM, we conducted a multivariable Competing Risk Analysis. The findings remained consistent after adjustment for age and diuretic dosages (Supplemental Table S3C, http://links.lww.com/HC9/B864). In those with peritonitis, isolated bacteria were *Staphylococcus spp*. (PeCa n=26 [45%], TIPS n=1 [10], followed by Enterococcus spp. (PeCa n=16 (28%), TIPS n=4 (40%); Supplemental Table S6C, http://links.lww.com/HC9/B864, Supplemental Figure S4, http://links.lww.com/HC9/B863). *Acinetobacter spp.* were only found in patients with PeCa (n=6 (10%); Supplemental Table S6C, http://links.lww.com/HC9/B864, Supplemental Figure S4, http://links.lww.com/HC9/B863). While ascites was the main reason for readmission in patients with TIPS (n=24 [46%]), patients with PeCa were most often readmitted due to renal impairment (n=21 [31%]; Supplemental with Table S5C, http://links.lww.com/HC9/B864).

## DISCUSSION

The onset of RA is a severe complication in patients suffering from ACLD.^5^ LTx should be considered in all patients with RA.^3^ If LTx is not available, TIPS implantation is considered the best alternative treatment option since it leads to a significant decrease in the portosystemic pressure gradient, the driving factor behind the formation of ascites.^1^ In case of contraindications against TIPS insertion, devices such as the ascites pump, or PeCa have been introduced to enable home-based treatment.^7^,^8^ Both devices have not been compared in terms of clinical complications, yet. Additionally, data comparing TIPS with any of the devices are scarce. To the best of our knowledge, this is the first study that directly compares TIPS, ascites pump, and PeCa. Notably, we observed a significantly higher incidence of peritonitis and device explantation in patients treated with PeCa compared to those treated with ascites pump. Furthermore, patients with TIPS implantation had lower incidences of clinical complications like hyponatremia, AKI, or rehospitalization compared to both devices.

Overall, mortality, AKI, and hyponatremia were comparable between the ascites pump and PeCa. Data from previous studies also implicate that these complications are most likely a result of the daily drainage volume and not attributable to the device per se.^7^ Higher drainage volumes result in a higher risk for electrolyte disturbances or AKI, even if the paracentesis volume is <5L.^7^,^20^ However, complications arising from device infections, ie, peritonitis or explanations, were considerably more frequent in patients with PeCa. A reason here might be the presence of an exposed external catheter in the PeCa group that may predispose to infections, given the high incidence of skin derived bacteria detected in the ascites cultures in those with PeCa. This is also underlined by the comparable peritonitis incidence between ascites pump and TIPS patients. Still, peritonitis incidence and rate of device explantations were comparably high in our PeCa cohort. A study from Macken et al^9^ observed peritonitis in only 6% of the included patients within 12 weeks after PeCa implantation. In their study, 7% of patients had a history of SBP (spontaneous bacterial peritonitis), while in our study, half of the analyzed PeCa patients experienced previous episodes of SBP. This might indicate a higher overall susceptibility for peritonitis.^21^ A previous study from our center did not observe significantly different incidences of SBP in patients with PeCa compared to patients undergoing repeated LVP.^12^ In this context, there was a numerically lower 90-day SBP incidence in patients treated with ascites pump compared to LVP in a prospective randomized controlled trial by Bureau et al.^8^ Given the repeated puncturing of the peritoneal cavity, LVP may also be a mechanism that could potentially introduce pathogens to the peritoneal cavity. This might explain the higher SBP incidence in patients treated with PeCa compared to ascites pump, but not between patients treated with PeCa and LVP. To address the high incidence of infection-associated device explantations, new PeCa have been introduced, which feature antimicrobial silver particles. First data implicate that these devices can lower the incidence of device infections.^22^ Hence, future studies need to compare the respective devices to determine whether the ascites pump remains associated with a lower incidence of infections and device explantation.

In clinical practice, the decision to implant an ascites pump over a PeCa is often based on the survival prognosis of the patient. Given the higher costs of the device, a minimum life expectancy of 6 months is usually required if treatment with the ascites pump is considered. Both options can be used to sufficiently control ascites-associated symptoms.^9^,^23^,^24^ Of note, there is some data implicating that both devices can be used as “bridge to transplant” procedures.^12^,^25^ PeCa implantation and explantation area relatively uncomplicated and easy to perform procedure which are well tolerated, even by severely compromised ACLD patients.^12^ For ascites pumps, device explantation has to be performed under general anesthesia. Furthermore, dedicated care services can also take care of the device and perform the drainage in a home-based setting. The ascites pump is overall less prone to external infections since it has no external pipes or tubes. However, it requires charging at least once or twice per day, depending on the output volume. This is typically not done by ambulatory care services, presenting a limitation if the patient or a family member cannot charge the device. Importantly, drainage volume can be easily adjusted in PeCa, while the ascites pump needs programming which is often performed in the implanting center, leading to readmissions. The main idea behind devices like the ascites pump and PeCa is to enable home-based treatment and symptom control, which both devices can achieve.^8^,^11^,^23^,^26^ Moreover, another treatment goal would be to prolong the time of a patient out of the hospital for as long as possible. Rehospitalization rates were high in patients with both devices, especially within the first 100 days after implantation. Ultimately, the decision of which device is offered to the patient has to be discussed with regard to the individual clinical fitness and prognosis in a case-by-case setting and, importantly, also with the patient.

Patients with devices had significantly higher incidences of AKI and hyponatremia compared to patients with TIPS. This is well in line with other studies. Will et al^10^ showed significantly higher incidences of hepatorenal syndrome in a small cohort of patients with an Alfapump compared to patients with TIPS​​​​​​. Solà et al^27^ observed significant drops in the glomerular filtration within the first 6 months after ascites pump insertion. Since TIPS targets the pathomechanism behind ascites, other complications that arise directly or indirectly from ascites drainage occur less frequently.^28^ Importantly, nutrients such as albumin or electrolytes are lost through ascites drainage.^29^ This problem does not occur in patients with TIPS as ascites production is effectively controlled after TIPS insertion. Additionally, TIPS implantation also improves other important factors, such as the hyperinflammatory state, which is present in patients with ACLD and RA.^30^,^31^ This is further underlined by the consistent improvement in nutritional status, muscle mass, and also quality of life after TIPS placement.^32^,^33^ Interestingly, while complications and rehospitalization rates were significantly higher in those with devices compared to TIPS implantation, LTx-free survival within 1 year did not differ. Since we only included patients with RA, ie, patients with highly progressed ACLD, the prognosis is limited in general, even after TIPS insertion. Nonetheless, our data support the use of TIPS over devices in patients with liver cirrhosis and RA since overall complications are significantly decreased.

This study has important limitations. First, the retrospective study design may have led to complications and adverse events going undetected despite our best efforts to ensure high data quality. Furthermore, patients with relatively good clinical fitness were selected for ascites pump, therefore selection bias is to be expected. Moreover, all patients with devices had underlying contraindications for TIPS. Even if we matched for important parameters via PSM, we have to assume that patients with devices are overall “sicker” as they may experience a far more progressed ACLD. Nonetheless, baseline characteristics and LTx-free survival were comparable after matching which at least implicates comparability to some degree. Last, limited sample sizes are one of the largest limitations of this study. However, with the PSM approach, we chose only a few patients from the ascites pump cohort had to be excluded from further analysis.

In conclusion, our study indicates that complications such as hyponatremia or AKI were comparable between patients with either ascites pump or PeCa, while the incidence of peritonitis and device explantations was higher in those with PeCa. TIPS was associated with significantly less clinical complications and rehospitalization compared to both devices and should therefore remain the SOC to treat RA, if LTx is not available.

## Data Availability

The data that support the findings of this study are available from the corresponding author upon reasonable request. Sarah L. Schütte: conceptualization, data curation, investigation, formal analysis, methodology, writing—original draft, writing—review and editing; Anja Tiede, Jim B. Mauz, Hannah Rieland, and Martin Kabelitz: data curation, investigation, writing—review and editing; Robin Iker: formal analysis, writing—review and editing; Nicolas Richter, Bernhard Meyer, and Benjamin Heidrich: resources, writing—review and editing; Heiner Wedemeyer: resources, writing—review and editing; Benjamin Maasoumy: conceptualization, formal analysis, methodology, project administration, resources, supervision, writing—review and editing; Tammo L. Tergast: conceptualization, data curation, investigation, formal analysis, methodology, project administration, supervision, resources, writing—original draft, writing—review and editing; All authors approved the final draft submitted. Sarah L. Schütte, Anja Tiede, Jim B. Mauz, Hannah Rieland, and Martin Kabelitz were supported by the “KlinStrucMed” program of the Hannover Medical School and/or by the “Else-Kröner-Fresenius Stiftung.” Robin Iker was supported by the “StrucMed” program of the Hannover Medical School. Heiner Wedemeyer consults and receives grants from Abbott. He is on the speaker bureau and receives grants from Biotest. He consults and is on the speaker bureau for Gilead. He consults for Bristol-Myers-Squibb, Hoffmann-La Roche, GlaxoSmithKline, Janssen, Roche, and Vir Biotechnology. Benjamin Maasoumy reports lecture and/or consultant fees from AbbVie, Fujirebio, Gilead, Luvos, MSD, Norgine, Roche, W. L. Gore & Associates. He received research support from Altona, EWIMED, Fujirebio, and Roche. The remaining authors have no conflicts to report.
